# Exploring Bracing Adherence in Ponseti Treatment of Clubfoot: A Comparative Study of Factors and Outcomes in Uganda

**DOI:** 10.3390/ijerph20146396

**Published:** 2023-07-19

**Authors:** Marieke Dreise, Catherine Elkins, Moses Fisha Muhumuza, Henry Musoke, Tracey Smythe

**Affiliations:** 1 MiracleFeet, Chapel Hill, NC 27514, USA; catherine.elkins@miraclefeet.org; 2 CoRSU Hospital, Kisubi, Uganda; moses.muhumuza@corsuhospital.org; 3 National Clubfoot Program Uganda, Kisubi, Uganda; ncpu.feet@gmail.com; 4 International Centre for Evidence in Disability, Department of Population Health, London School for Hygiene and Tropical Medicine, London WC1E 7HT, UK; tracey.smythe@lshtm.ac.uk; 5 Division of Physiotherapy, Department of Health and Rehabilitation Sciences, Stellenbosch University, Cape Town 7602, South Africa

**Keywords:** clubfoot, Ponseti method, foot functionality, foot abduction brace, adherence

## Abstract

The Ponseti method of clubfoot treatment involves two phases: initial correction, usually including tenotomy; and bracing, to maintain correction and prevent relapse. Bracing should last up to four years, but in Uganda, approximately 21% of patients drop from clinical oversight within the first two years of using the brace. Our study compared 97 adherent and 66 non-adherent cases to assess the influential factors and effects on functional outcomes. We analyzed qualitative and quantitative data from clinical records, in-person caregiver interviews, and assessments of foot correction and functionality. Children who underwent tenotomy had 74% higher odds of adherence to bracing compared to those who did not undergo tenotomy. Conversely, children from rural households whose caregivers reported longer travel times to the clinic were more likely to be non-adherent to bracing (AOR 1.60 (95% CI: 1.11–2.30)) compared to those without these factors. Adhering to bracing for a minimum of two years was associated with improved outcomes, as non-adherent patients experienced 2.6 times the odds of deformity recurrence compared to adherent patients. Respondents reported transportation/cost issues, family disruptions, and lack of understanding about the treatment method or importance of bracing. These findings highlight the need to address barriers to adherence, including reducing travel/waiting time, providing ongoing education for caregivers on bracing protocol, and additional support targeting transportation barriers and household complexities.

## 1. Introduction

Clubfoot is a congenital foot deformity characterized by inward rotation of the heel and a twisted position of the foot, resulting in lifelong disability, pain, exclusion, and diminished quality of life if left untreated [1]. In 1999, Uganda was the first country in the world to implement a national clubfoot program using the Ponseti method and public health principles [2]. From Uganda this concept has spread across Africa and worldwide to many low- and middle-income countries (LMICs). Local and international non-governmental organizations (NGOs) now collaborate with numerous LMIC governments to expand quality comprehensive clubfoot services. These efforts often focus on increasing early detection and referral, building skills among clubfoot treatment providers, and ensuring children have access to high-quality care, including through provision of clinical oversight and follow-up especially during the lengthy maintenance phase. Many NGOs collaborate through the Global Clubfoot Initiative, pooling their forces and resources [3]. 

The National Clubfoot Program of Uganda (NCPU) is a cooperation between the NGO Comprehensive Rehabilitation Services of Uganda (CoRSU), the Ugandan Ministry of Health, and MiracleFeet, an international NGO supporting local initiatives and national clubfoot programs in more than 30 countries. NCPU supports 30 clubfoot clinics across Uganda, mainly in government hospitals. The program provides training and supervision to providers in treatment skills, trains parent advisors and other health workers in early detection and referral, provides free materials, such as plaster of Paris for casting and foot abduction braces where needed, and raises awareness about the treatability of clubfoot. With an incidence of around 1.2 per thousand live births [4,5], an estimated 2100 babies are born in Uganda each year with unilateral or bilateral clubfeet. 

The Ponseti method is the global gold standard for clubfoot treatment. It involves a correction phase that includes weekly manipulations and casting of the affected foot using plaster casts, typically ranging from four to six casts per child, followed by a percutaneous Achilles tendon tenotomy and a final cast. This phase achieves excellent results in realigning the foot, with an estimated success rate of 95% when treatment starts in the first year of life [6,7,8,9,10,11]. The correction phase is followed by the maintenance phase, which involves the use of a foot abduction brace to maintain the corrected alignment of the foot while the child’s musculoskeletal frame matures. According to the protocol, parents are required to keep the child’s feet regularly braced with shoes fixed to a bar that holds each foot in 70° abduction and 10–15° dorsiflexion [12]. Initially, the child wears the brace for 23 h per day for the first three months, and, subsequently, when sleeping until around 4 to 5 years of age. Following this protocol reduces recurrence rates [12,13,14]. However, non-adherence to the bracing regime can lead to recurrence of the deformity, resulting in poor functionality, pain, and other complications, which are more challenging to correct in older children [15,16,17].

Adherence to the bracing protocol through a sufficient maintenance period, therefore, remains a continual concern. The aim of this study was to examine factors that influence adherence to the bracing protocol and to investigate the impact of adherence to the bracing protocol on functional outcomes in Uganda. Understanding the factors that influence adherence can help identify barriers and develop interventions to improve adherence rates, which can ultimately lead to better functional outcomes for affected children. 

## 2. Materials and Methods

### 2.1. Study Design, Participants, and Setting

The mixed-method study consists of a retrospective cohort study, with case comparison, that includes reviewing clinical records, conducting examinations of the child’s affected feet or foot, and administering a caregiver survey with closed- and open-ended questions to collect household demographic information and qualitative perspectives on clubfoot-related experiences. Two comparable patient groups, adherent and non-adherent, were constructed based on whether caregivers adhered to clinical oversight (appointments) through the bracing protocol. Unilateral and bilateral clubfoot cases were included in the study. To be eligible for inclusion in the study, a patient’s case record had to meet the following criteria:

- Enrolled for treatment at one of the six major clubfoot clinics in Uganda, across the four regions of the country.
- Recorded birthdate between 1 March 2018 and 1 March 2021, so children would be at least walking age (approximately 1.5–4.5 years old) before data collection.
- Idiopathic clubfoot diagnosis (i.e., cases with postural, syndromic, and secondary diagnoses were excluded).
- Achieved full correction by end of the casting phase, according to clinic records of Pirani scores (measure of severity of clubfoot deformity).
- Started the bracing protocol (first bracing visit) before the first birthday.

An additional criterion for assignment to the non-adherent group wasa minimum period of at least six months since the child’s last missed appointment. 

Patient lists were constructed as per the inclusion and assignment criteria above to comprise the sampling frame. Phone numbers were extracted from electronic patient records and the identity of the primary caregiver, parent, or guardian was confirmed by a phone call to invite the caregiver and child to participate in the study. Participation included evaluation of the child’s treatment outcome and an opportunity to provide feedback in the caregiver survey.

A total of 178 adherent parents or caregivers were contacted by phone and informed about the study, of which 97 (54%) agreed to participate. Similarly, among the non-adherent parents or caregivers contacted by phone (n = 139), 66 (47%) agreed to participate. In total, 163 children participated in the study, with 97 (59.5%) adhering to treatment in the maintenance phase and 66 (40.5%) having not attended a clinic appointment for at least six months after a missed appointment.

Research associates collected primary data for the study through interviews and clinical examinations after a one-day training provided by the study team. The study team selected experienced Ponseti providers to conduct the clinical exams and parent advisors to conduct caregiver interviews. The criteria for selecting providers included demonstrated excellence in the Ponseti method, completion of supportive supervision training, and at least four years of experience mentoring and supervising other Ponseti providers. Each selected provider conducted clinical examinations in clinics where they normally do not provide supportive supervision. The providers recorded results of the clinical examination of each affected foot using observation and examination, following the scoring systems as described in the next paragraph.

The study team also selected experienced parent advisors to interview the caregivers, who conducted their data collection in clinics outside their normal scope of activities. Parent advisors are volunteers trained by NCPU to support parent education and outreach. The questionnaire, based on the survey tool used in Bangladesh [18] and adjusted, with open questions to give parents the opportunity to freely choose their own replies, included sections on household (non-parent caregivers were asked about the education and employment of the parents, while answering other household questions (location, other adults in the household, availability of help with other chores when traveling with the child for clinic visits) with reference to their own households), demographics (marital status, education, employment, number of siblings, etc.), experiences managing the child’s clubfoot treatment (travel, challenges, etc.), and perspectives on the child’s condition and treatment (personal and family response to clubfoot and ways the household managed, or did not manage, through the challenges). Four scoring systems that are designed to assess the child’s functional mobility were also included.

The questionnaires were available, and interviews conducted in four languages: English, Luganda, Runyankole, and Luo. Responses were initially recorded by hand on paper. 

In conducting this study, we recognize the significance of reflexivity and its potential influence on the data collection process. The caregiver interviews were conducted by parent advisors who were experienced volunteers trained by the NCPU to provide parent education and outreach support. It is important to note that these parent advisors, both male and female, carried out the interviews outside their usual scope of activities in clinics and in clinics where they do not volunteer. Their involvement in the study as both interviewers and individuals with personal experiences of clubfoot care may have introduced biases that could impact the data collection and subsequent analysis. We aimed to mitigate these potential biases by providing comprehensive training to the parent advisors on interview techniques and maintaining ongoing communication throughout the data collection process, and assigning each to conduct data collection activities in different clinic locations. Furthermore, efforts were made to establish a supportive and collaborative environment, encouraging open dialogue and reflection to address any potential biases that may have arisen. These reflexivity considerations enhance the transparency and validity of our study, contributing to a nuanced interpretation of the findings.

### 2.2. Assessing Functionality 

Different scoring systems are available to measure foot function [19,20,21], however, some tools entail a lengthy process and need complicated measuring tools. For our setting we selected tools that are robust, yet practical:

- The Bangla clubfoot tool [22], developed in Bangladesh, drawing on existing validated instruments, is validated, with good reliability, and is easy to use. Application of this tool includes gathering data on the parents’ observation of the child and observes the child’s gait directly along with a clinical foot examination.
- The Assessing Clubfoot Treatment (ACT) tool was developed with 18 experienced clubfoot practitioners and trainers from ten countries in Africa, using the Delphi method to reach consensus on the highest-rated or valued outcomes: a plantigrade foot, ability to wear a normal shoe, parent satisfaction, and absence of pain [23]. This tool was used in an audit in Zimbabwe and proved ‘simple to administer, had excellent observer agreement and good sensitivity and specificity in identifying children who need further intervention’ [24].
- The Oxford Ankle Foot Questionnaire, a patient reported outcome measure, the parent version, gathers more dimensions of the parents’/guardians’ opinion about the foot of their child [25], with 15 questions versus four or five.
- For patients diagnosed with a relapse during the clinical examination portion of data collection, Ponseti providers serving as research associates then applied the classification of Bhaskar and Patni [26] to the case. This classification consists of 5 relapse patterns categorized in 3 grades:
  - Grade IA: Decrease in ankle dorsiflexion from 15° to neutral.
  - Grade IB: Dynamic forefoot adduction or supination of foot. Both these are flexible relapse patterns.
  - Grade IIA: Fixed equinus of any degree (passive correction to neutral not possible).
  - Grade IIB: Fixed adduction of forefoot and midfoot (fixed lateral curvature). In this pattern there is fixed deformity in one plane, e.g., sagittal (equinus) or horizontal (adduction).
  - Grade III: Two or more fixed deformities (fixed equinus and adduction and cavus or complete relapse of clubfoot deformity with heel varus, ankle equinus, midfoot cavus, and forefoot adduction).

### 2.3. Data Collection, Management, and Analysis 

The primary data for the study were collected at the selected NCPU clinics in August and September 2022. Parents/guardians (primary caregivers) who consented and children who were enrolled as patients for clubfoot treatment in one of the selected NCPU clinics, were convened at the clinic for the data collection session. During data collection, experienced clubfoot care providers serving as research associates examined affected feet for functionality and relapse and recorded their clinical findings. Parent advisors, also research associates, interviewed the primary caregiver in-person during the same visit, recording their responses on paper.

Researchers supplemented the survey and observational data collected on paper with information extracted from medical records into an electronic spreadsheet. The additional information incorporated in the study data set for analysis included the clinic attended, birth date, sex, unilateral or bilateral condition, Pirani score at first visit, number of casts, and tenotomy status. 

Quantitative data were imported for analysis in Stata 14.2 (StataCorp 4905, Lakeway Drive College Station, TX 77845, USA). We defined the variable of recurrence based on the Bhaskar and Patni classification [26], specifically focusing on grades II and III. We chose these classifications because they indicate a higher likelihood of necessitating additional clinical intervention due to recurrence. Descriptive statistics, proportions, and regression models were used to compare household, clubfoot, and treatment characteristics of children who adhered to bracing and those who did not. Statistical significance for the case series was set at the 95% confidence level. We used a logistic regression model to test for the influence of the child’s sex, bilateral versus unilateral clubfoot, age at first brace, starting Pirani score, above- or below-average number of casts, socioeconomic status, and tenotomy status on adherence to bracing. The model was fitted for the outcome of adherence to bracing on an individual level as children with bilateral clubfeet had the same number of casts applied per foot to enable both tenotomies to be performed on the same day. For qualitative data, the open-ended survey responses were thematically analyzed.

## 3. Results

Similarities between the characteristics of children whose caregivers adhered to the bracing protocol and those who did not include household location (rural or town) and number of children in the household; parents’ education, occupation, and marital status; and patient’s sex (Table 1). 

The characteristics of the children’s clubfoot conditions retrieved from clinical records likewise show similarities across the two groups, including sex distribution, feet affected by clubfoot, Pirani score at first visit, number of casts during the correction phase, or age at the initiation of bracing. However, in terms of treatment, a greater proportion of children whose caregivers adhered to the bracing protocol had received tenotomies (91.8% vs. 75.8%, *p* = 0.0045) (Table 2). 

### 3.1. Outcome of Foot Functionality

The tools used in this study to assess foot functionality consistently yielded outcomes that demonstrated poorer foot functionality among children whose caregivers did not adhere to bracing compared to those who did. Table 3 displays the scores and sub scores of the Bangla and Assessing Clubfoot Treatment (ACT) and the Oxford Ankle Foot Questionnaire tools. The tools cover similar topics with some variations, and consistency in results using any of the three is a helpful finding, suggesting that further research can select the most appropriate tool for a population or the study design with some assurance that any of these tools are likely to yield comparable data and findings.

For instance, the Oxford series asks the respondent to “Think about your child in the last week” before asking 15 questions, while the ACT asks only four questions in the present tense. The Bangla tool has no timeframe and asks the caregiver five questions with additional observations of the child’s mobility and a clinical examination. Questions are similar but not identical, e.g.:

- Happy with child’s feet? (Bangla)
- How satisfied are you with your child’s foot? (ACT)

With respect to footwear:

- Can your child wear shoes of your/their choice? (ACT)
- Does child wear shoes of choice? (Bangla)
- [Thinking about last week] Has your child’s foot or ankle stopped them wearing any shoes they wanted to wear? (Oxford)

And activities:

- [Thinking about last week] Has your child’s foot or ankle stopped them joining in with others in the playground? (Oxford)
- Does child play with others? (Bangla)

Pain-related questions:

- Does your child complain of pain in their affected foot? (ACT)
- Does child have pain? (Bangla)
- [Thinking about last week] Has your child had pain in their foot or ankle? (Oxford)

According to the Bhaskar and Patni relapse grading system, there were 5 patients with grade II relapse (2 bilateral) and 20 patients with grade III relapse (12 bilateral). 

Among the 25 patients with grade II or grade III relapse, 9.3% (n = 9) were found to be adherent to the treatment protocol and 24.2% (n = 16) were non-adherent. There was a statistically significant difference in relapses between the adherent and non-adherent groups (*p* = 0.0091), meaning that the observed disparity in adherence to the treatment protocol among patients with grade II or grade III relapse is unlikely to have occurred by chance and indicated a meaningful distinction between the adherent and non-adherent groups.

Adhering to bracing for a minimum of two years was linked to improved outcomes, as non-adherent patients experienced 2.6 times the odds of deformity recurrence compared to adherent patients.

### 3.2. Factors Influencing Adherence to Bracing and the Role of Tenotomy

A logistic maximum likelihood regression analysis revealed that even after adjusting for other variables within the model, the relationship between tenotomy performed and adherence remained, with an adjusted OR of 0.26 (95% CI [0.08–0.87]) (Table 4) This suggests that children who underwent tenotomy were estimated to have 74% higher odds of adhering to bracing compared to those who did not undergo tenotomy. Additionally, endogenous factors played a significant role in influencing adherence behavior in the context of the study. Children from rural households and with longer travel times to the clinic were more likely to be non-adherent to bracing (AOR 1.60 (95% CI: 1.11–2.30)) compared to those who do not have these factors. The odds of non-adherence were 60% higher in children from rural households and with longer travel times compared to those without these factors.

### 3.3. Parent Survey

A qualitative analysis of the questionnaires revealed that parents cited financial and transportation issues as the primary reasons for not coming back for follow-up appointments (Table 5). This included increased costs and uncertainties during lockdowns, household finances being depleted due to life events or other medical expenses, and household relocations farther from the clinic. Other themes that emerged included complications in caring for other children, marital issues or non-parent caregivers, a poor clinic experience, and lack of understanding about the importance of bracing as parents thought their child was doing fine. These findings highlight the various barriers faced by parents in adhering to the bracing protocol, including financial constraints, logistical challenges, and lack of awareness about the importance of continued care, which need to be addressed to improve adherence rates and treatment outcomes.

The reasons for difficulties in attending clinic given by parents of children who adhered and who did not adhere to treatment centered around the cost, time, and availability of transport. These factors were more prevalent among non-adherent respondents, with 87.5% compared to 66.0% of adherent respondents experiencing them. 

Parents reported facing various challenges during different phases of the Ponseti method for clubfoot treatment. During the casting phase, issues related to the weight of the plaster and transport were mentioned. During the tenotomy phase, parents reported concerns about pain, sleepless nights, and the need for special attention. Parents reported bracing phase challenges such as getting the child to cooperate, the child crying, or the child removing the brace. When asked about if there was a phase of the treatment that parents found particularly challenging and why, only 23.6% of respondents whose children adhered to bracing identified the bracing phase as challenging, compared to 50.8% of respondents whose children who did not adhere. A total of 152 respondents mentioned facing at least one challenge across the different phases. Some parents also mentioned challenges related to the cost of transportation, expressing an inability to afford it (Table 6). These findings underscore the practical and emotional difficulties experienced by parents throughout the different phases of clubfoot treatment. They emphasize the need for additional support and interventions to address these challenges and improve treatment adherence.

## 4. Discussion

This study found that adherence to bracing in the maintenance phase of clubfoot treatment was influenced by several factors. Children who underwent tenotomy had a higher likelihood of adhering to bracing, while living in a rural location and longer travel times to the clinic were associated with higher odds of non-adherence. Financial and transportation challenges were significant barriers to adherence, emphasizing the importance of additional support and awareness to improve treatment outcomes.

Our findings align with previous research. Dobbs et al. [16] found that 41% of patients in their study did not wear the brace according to the protocol, and among non-adherent patients, 76% (n = 16/21) experienced recurrence. This study found that recurrences were not related to the severity of the clubfoot, age of presentation, or previous treatment. Similarly, Goksan et al. [27] found that brace non-adherence was the most important risk factor for recurrence, with a 32% recurrence rate for non-adherent patients versus 9% for adherent patients. These findings are consistent with other studies worldwide [28,29,30,31,32,33].

Studies suggest that patients who achieve successful correction post-tenotomy but fail to adhere to the bracing protocol during the maintenance phase remain susceptible to recurrence of the deformity and poor functional outcomes [15,33]. A survey conducted by the Global Clubfoot Initiative in 2015 revealed that 47% of patients were completing less than two years in bracing [34]. In Uganda, around 21% of NCPU patients within two years of bracing are categorized as dropouts because they have not been seen by a provider for at least six months after a missed appointment (clinical records, April 2023).

### 4.1. Consequences of Dropping out of Treatment

The consequences of dropping out of treatment for clubfoot in Uganda are also similar to findings from other studies. McElroy et al.’s ethnographic study conducted in Uganda [1] found that all participants, regardless of their beliefs, recognized the importance of clubfoot treatment, yet significant barriers hindered their ability to continue treatment, leading to high dropout rates during the casting and bracing phases. The Bangla, ACT, and Oxford assessment scores in our study consistently demonstrated the consequences of discontinuing treatment during the bracing phase, affecting both the child’s outcomes and the caregiver’s assessment of and satisfaction with treatment results.

Moreover, our grading according to Bhaskar and Patni shows that recurrence (defined as grade II and III relapses) occurred 2.6 times as often in children that did not adhere to bracing compared to those who did. The fixed deformities of grade II and III relapses can regularly only be solved by renewed casting plus surgical intervention, which is not readily available and is costly in Uganda.

### 4.2. Reasons for Non-Adherence

We did not find significant differences in demographic variables (marital status, level of education, parents’ occupations, and number of other children in the household) between adherent and non-adherent respondents. Factors related to the laterality of the clubfoot (uni/bilateral), severity of the deformity (Pirani score at first visit), number of casts, and age at first brace also were not significantly different between the two groups.

#### 4.2.1. Level of Education

Although our study did not find any relationship between level of education and adherence, Dobbs et al. [16] concluded that there is a link between education level (high school and less) and recurrence in the United States. Pigeolet et al. [35] in their review of 19 studies examining the impact of socioeconomic factors on non-adherence found no significant relationship with education. However, they noted that some studies were able to link a lack of knowledge about the Ponseti treatment and clubfoot in general to drop-out (Evans et al. [17]), while Kazibwe et al. [36] found that 79% of parents were unaware of their responsibility to adhere to a bracing protocol after the correction phase, although this lack of knowledge did not influence adherence.

#### 4.2.2. Poverty

Poverty emerged as the primary reason for dropping out of clubfoot treatment in our study. Despite the treatment being free of charge in Uganda, lack of financial resources presents a significant barrier to accessing treatment. According to the World Bank’s Poverty and Equity brief on Uganda, 56 percent of the population is moderately food insecure and 15 percent severely food insecure. Many families are forced to prioritize survival over seeking treatment for a non-life-threatening deformity, especially those in rural areas and extreme poverty, who struggle with rising prices and limited access to an adequate food supply [37].

In terms of monetary poverty, 30.1% of the Ugandan population lives below the poverty line of USD 1.90 per person per day (2019 data, Uganda Bureau of Statistics, UBOS). However, monetary poverty alone does not fully capture the extent and depth of deprivations experienced by children and adults, and, therefore, multidimensional poverty is considered a better measure of poverty. Multidimensional poverty takes into account household characteristics, geographical location, and individual characteristics [38]. Nationally, an average of 47% of households experience multidimensional poverty, with rural areas reporting a higher rate of 55%, more than double that in urban areas at 23%. This finding corroborates our survey results, which indicate that living in rural areas poses challenges to adhering to treatment and attending regular follow-up visits due to long distances, high transportation costs, and time away from home. Furthermore, our study reveals a significant relationship between living in a rural area, travel time to the clinic, and non-adherence to bracing.

Notably, the issue of poverty, including transportation costs and time away from home, has been consistently highlighted in previous studies as a barrier to accessing clubfoot treatment in LMIC [35,39].

#### 4.2.3. Tenotomy

In our study, we identified an association between the absence of a tenotomy procedure and non-adherence to the bracing protocol. The correction of equinus, where the foot points downward, is the last deformity corrected in the Ponseti method. If equinus remains, it will be difficult to fit the foot into a brace. Equinus is corrected by tenotomy. Cohen et al.’s study [40] reported a tenotomy rate of 61.1% and suggested that a higher tenotomy rate could lead to a lower recurrence rate among their patients. They did not initially perform tenotomy in cases when the foot was in a neutral position, but revised their approach based on their study findings, and now consider tenotomy as a precondition for reducing the recurrence rate. Hosseinzadeh et al. [41] also reported that patients with residual equinus, defined as neutral or less then neutral dorsiflexion after a tenotomy, had a 64% likelihood of requiring additional surgical procedures for a recurrence, compared to patients with around 15° dorsiflexion. While there is limited literature on the relationship between the absence of tenotomy and non-adherence to bracing, we recommend further research to investigate this association. One possible explanation is that failure to achieve 15° dorsiflexion in the correction phase makes it challenging and uncomfortable to fit the foot into the brace. This can lead to increased resistance, protests, and crying by the child, making it difficult for caregivers to consistently apply and maintain the brace as per the protocol, resulting in reduced adherence to the recommended bracing period. Although the sample size in our study is too limited to draw definitive conclusions, it is worth noting that most children in the study who did not undergo tenotomy were over 3 years of age at the time of data collection. Moreover, recent data from our study shows an increased tenotomy rate of 82.5% in 2023. While this program has partially addressed this issue of low tenotomy rates, other programs can benefit from a better understanding of these relationships and mechanisms to increase adherence.

### 4.3. Recommendations to Increase Adherence to the Bracing Protocol

Despite establishing many clinics across the country and supporting 30 clinics in 2023, study respondents commonly cited long distances, travel time, and transportation costs as the main reasons for non-adherence. Addressing this issue requires collaboration with the Ministry of Health to establish clinics closer to patients’ homes while ensuring quality standards. GCI’s RunFree 2030 global strategy [42] strongly recommends that a clubfoot clinic enrolls at least 24 new patients per year in order to maintain providers’ practice of Ponseti technique skills. Therefore, the establishment of new clinics must strike a balance between proximity to patients’ homes and ensuring the quality of treatment.

Parents also expressed concerns about the long waiting times at the clinics, which make it difficult to return home in a timely manner. Providers should be present and prepared with all necessary supplies to start their clinics on time and ensure efficient clinic operations. Additionally, the program could advocate for more trained providers in existing clinics to improve patient flow and significantly reduce waiting times. Other solutions could be initiating additional clinic days, introducing a ‘brace’ day, or decentralizing brace management to health centers closer to the patients. The Somali Clubfoot Program supported by ICRC/MiracleFeet is an excellent example of effective decentralization, where selected health centers’ staff are trained in brace management and receive supervision from the clubfoot clinics.

Providing parents who understand the importance of bracing with an extra brace and providing instructions for when to change to a larger size could be another option. With the increasing use of WhatsApp among parents, video calls can be used to check the fit and condition of the brace. However, above all, building a good relationship with parents, and adopting a supportive approach that addresses their individual concerns while ensuring their understanding of the critical importance of bracing is essential. According to the Agency for Health Care Research and Quality [43], Kessels et al. [44] found that up to 80% of the medical information provided to patients was forgotten immediately, and Anderson et al. [45] described how half of the information remembered by patients was incorrect. To address this, the Teach-Back method can be employed to ensure that parents really understand what is explained to them. A brief explanation of the Teach-Back method is available on YouTube [46].

The National Clubfoot Program of Uganda cannot solve poverty, which is an underlying factor exacerbating other barriers. However, NCPU has improved brace adherence over time through adopting various measures, reducing the dropout rate among patients in their first 2 years of bracing from around 40% five years ago to around 20% currently (21%, April 2023). The NCPU initiated clubfoot clinics in new facilities distributed more widely across the country and now supervises 30 clinics in all regions of Uganda. A transport subsidy system was established for the poorest patients. Parent education has been improved through recruiting and training volunteer parent educators who commit to being present every clinic day to advise and support parents who may be confronting bracing for the first time, or who are experiencing challenges in this phase. These parent educators perform home visits when parents do not return for appointments and do not show up after repeated phone calls. Often the parent educators already know of social problems at home (alcoholism, severe poverty, marital issues, one partner not supporting treatment, etc.) as they have built up a relationship with a parent, mainly the mothers. Discussing the benefits of the treatment with both parents and other relatives, assisting with transport subsidies, asking the community, local social workers, church leaders, etc., to step in and assist/support the family to deal with their problems may all help in motivating parents to come back. The CAST smart phone system for patient records, with a feature for showing missed appointments connected with telephone numbers of the parent, has improved contacting parents soon after they do not show up for a check-up. A list of additional recommendations can be found in the Supplementary Materials.

### 4.4. Limitations

Aligned to the program model and experience, we expected non-adherence to be more likely for children from socioeconomically disadvantaged households, those living at greater distance from the clinic, and those presenting with more severe clubfoot or at a later age. The study inclusion criteria, however, required children diagnosed with non-idiopathic clubfoot (secondary and postural) who started bracing before reaching 1 year old. Additionally, most NCPU clinic patients come from disadvantaged and rural households. These elements of the study design limit our ability to detect variations in these factors within our sample. A further limitation of our study is the participation rate, with slightly more than half (51%) of the caregivers we were able to reach agreeing to participate. This introduces a potential selection bias, as the sample may predominantly represent the relatively better-off households currently living in closer proximity to the clinics. As a result, the interpretation of clinical and functional outcomes may be influenced, and caution should be exercised when generalizing the findings to a broader population of parents of children with clubfoot. 

Furthermore, while we classify parents as adherent based on their regular clinic follow ups, we cannot be entirely certain that they are consistently using the brace every night as prescribed. We also assume that dropouts are not adhering to treatment, but it is possible that some parents who do not attend follow-up visits may follow the prescribed protocol. To mitigate these limitations, we explored these factors in the qualitative survey.

## 5. Conclusions

Our study highlights the importance of adherence to bracing in the maintenance phase of clubfoot treatment. Children who underwent tenotomy had significantly higher adherence rates, while living in rural areas and longer travel times to the clinic were associated with higher non-adherence. Financial and transportation challenges were major barriers to adherence. Recurrence of deformity was more common in non-adherent patients. Poverty emerged as the primary reason for dropping out of treatment. Addressing these challenges requires establishing clinics closer to patients’ homes, reducing waiting times, increasing provider capacity, and improving communication with parents. Further research is needed to explore the relationship between tenotomy and adherence, as well as the impact of education on adherence rates. Improving adherence is essential for enhancing treatment outcomes for clubfoot patients.

## Figures and Tables

**Table 1:** Cohort and household characteristics.

Household and Patient		Adherent Children n (%)	Non-Adherent Children n (%)	Total
Total		97	66	163
Household site (n = 153)	Town	34 (38.2%)	15 (23.4%)	49 (32.0%)
	Rural	55 (61.8%)	49 (76.6%)	104 (68.0%)
Total children in household (n = 160)	1	13 (13.7%)	4 (6.2%)	17 (10.6%)
	2–3	35 (36.8%)	24 (36.9%)	59 (36.9%)
	4–5	22 (23.2%)	14 (21.5%)	36 (22.5%)
	>5	25 (26.3%)	23 (35.4%)	48 (30.0%)
Parents’ education, highest level attended by either parent (n = 161)	No schooling	4 (4.2%)	0 (0.0%)	4 (2.5%)
	Primary school only	23 (24.2%)	20 (30.3%)	43 (26.7%)
	O level	34 (35.8%)	26 (39.4%)	60 (37.3%)
	A level	7 (7.4%)	6 (9.1%)	13 (8.1%)
	Tertiary	5 (5.3%)	6 (9.1%)	11 (6.8%)
	University	22 (23.2%)	8 (12.1%)	30 (18.6%)
All parents’ occupations (n = 298)	Unemployed	13 (7.4%)	13 (10.7%)	26 (8.7%)
	Unskilled or manual labor	48 (27.3%)	35 (28.7%)	83 (27.9%)
	Clerical, sales, and/or services	56 (31.8%)	31 (25.4%)	87 (29.2%)
	Skilled production	17 (9/7%)	16 (13.1%)	33 (11.1%)
	Skilled trades, crafts	24 (13.6%)	10 (8.2%)	34 (11.4%)
	Professional or technical	18 (10.2%)	17 (13.9%)	35 (11.7%)
Parents’ marital status	Married or live together	78 (81.3%)	51 (77.3%)	129 (79.6%)
	Divorced/separated	10 (10.4%)	9 (13.6%)	19 (11.7%)
	Widowed	3 (3.1%)	1 (1.5%)	4 (2.5%)
	Never married or living together	5 (5.2%)	5 (7.6%)	10 (6.2%)

Note: Child’s sex was extracted from patient records. Respondents provided information on other items; non-parent and single-parent respondents may have incomplete or incorrect knowledge of the (other) parent’s or parents’ educations and occupations.

**Table 2:** Clubfoot and treatment characteristics.

		Adherent Children n (%)	Non-Adherent Children n (%)	Total
Total		97	66	163
Patient’s sex	Male	79 (81.4%)	50 (75.8%)	129 (79.1%)
	Female	18 (18.6%)	16 (24.2%)	34 (20.9%)
Feet affected by clubfoot	Unilateral	42 (43.3%)	31 (47.0%)	73 (44.8%)
	Bilateral	55 (56.7%)	35 (53.0%)	90 (55.2%)
Pirani score at first visit, all affected feet	0–1.0	5 (3.3%)	1 (1.0%)	6 (2.4%)
	1.5	0	0	0
	2.0–6.0	148 (96.7%)	100 (99.0%)	248 (97.6%)
Number of casts	Fewer than 4	29 (29.9%)	16 (24.2%)	45 (27.6%)
	4 to 6	44 (45.4%)	29 (43.9%)	73 (44.8%)
	More than 6	24 (24.7%)	21 (31.8%)	45 (27.6%)
Tenotomy	yes	89 (91.8%)	50 (75.8%)	139 (85.3%)
	no	8 (8.3%)	16 (24.2%)	24 (14.7%)
Age at first brace	1–3 months	67 (69.1%)	44 (71.0%)	111 (69.8%)
	4–6 months	19 (19.6%)	13 (21.0%)	32 (20.1%)
	7–9 months	7 (7.2%)	4 (6.5%)	11 (6.9%)
	10–12 months	4 (4.1%)	1 (1.6%)	5 (3.1%)

Note: Data extracted from patient records.

**Table 3:** Bangla, ACT, and Oxford assessment scores.

Tool	Tool Category (Score Range)	Adherent Mean Score (95% CI)	Non-Adherent Mean Score (95% CI)
Bangla scores	Total (−11/11)	8.85 (8.4–9.3)	6.5 (5.5–7.4)
	Parent assessment (−5/5)	4.7 (4.5–4.9)	3.6 (3.1–4.2)
	Gait assessment (−4/4)	3.5 (3.3–3.7)	2.9 (2.6–3.2)
	Clinical examination (−2/2)	0.8 (0.6–1.0)	0.0 (−0.3–0.3)
ACT scores	Total score (0/12)	10.7 (10.3–11.2)	9.2 (8.4–10.0)
	Foot is plantigrade (0/3)	2.7 (2.5–2.8)	2.2 (1.9–2.4)
	No pain (0/3)	2.9 (2.8–3.0)	2.6 (2.4–2.8)
	Child wears shoes of choice (0/3)	2.7 (2.6–2.9)	2.4 (2.2–2.6)
	Parent satisfaction (0/3)	2.6 (2.5–2.7)	2.0 (1.8–2.3)
Oxford Ankle Foot Questionnaire *	Physical (0/24)	21.5 (20.7–22.3)	18.8 (17.3–20.3)
	Play (0/8)	7.9 (7.7–8.0)	7.5 (7.1–7.9)
	Emotional (0/20)	14.6 (14.0–15.2)	13.8 (12.8–14.7)

* Questions 9–10 of the Oxford Ankle Foot Questionnaire were dropped as the questions were related to school attendance; the children in our study were too young to go to school.

**Table 4:** Model of factors affecting non-adherence.

Predictor Variables	Univariate OR (95% CI)	*p*-Value	Multivariate AOR (95% CI)	*p*-Value
Child’s sex	0.71 (0.33–1.52)	0.38	0.85 (0.29–2.46)	0.77
Parents married or living together	0.83 (0.39–1.78)	0.63	0.77 (0.26–2.27)	0.63
Highest level of parents’ education	0.46 (0.19–1.10)	0.08	0.64 (0.18–2.35)	0.50
Parent occupation	1.11 (0.59–2.08)	0.74	1.20 (0.51–2.79)	0.68
Number of children in household	1.10 (0.97–1.26)	0.13	1.03 (0.87–1.23)	0.72
Rural household	2.02 (0.98–4.15)	0.06	0.64 (0.16–2.60)	0.53
Clinic travel time	1.18 (1.01–1.37)	0.04	1.00 (0.85–1.17)	0.97
Interaction: Rural and Travel time			1.60 (1.11–2.30)	0.01 *
Bilateral or unilateral	0.86 (0.46–1.62)	0.64	0.60 (0.24–1.44)	0.25
Pirani score (initial)	1.17 (0.87–1.57)	0.29	1.22 (0.84–1.77)	0.29
Number of casts	0.94 (0.50–1.78)	0.86	0.97 (0.41–2.29)	0.95
Tenotomy performed	0.28 (0.11–0.70)	0.01	0.26 (0.08–0.87)	0.03 *
Age at first brace	0.67 (0.86–1.13)	0.83	0.98 (0.83–1.16)	0.83

* = significant *p*-values.

**Table 5:** Difficulties encountered by parents concerning travel.

Issues	Frequency	Sample Comments
Time, distance, or time away from home	25	“It takes a long time to reach the hospital because of bad roads and heavy traffic”
Expenses or financial strain related to transport	62	“Sometimes the date of review reaches, when there is no money to pay transport and you miss out”
General hassles of transport, lack of access to transport	55	“Transport is hard to get, few matatus available.” “Transport costs change every day; the jam is always too much”
Specific mention of lockdown, pandemic conditions	11	“He was born during the covid period, so the high cost of transport or sometimes having to carry him to the clinic was tiresome”
Family challenges, household responsibilities	4	” Having a family with many children and transport cost increasing”
	Number of respondents mentioning at least one challenge: 113 Mean number of challenges mentioned: 1.4 (max = 4)	
“Difficult in covid, because I used to move on foot from [home] to [clinic], even going back. Difficult because I live alone to care for my children and my relatives and transport becomes a problem.” “Difficult because my baby was only 2 weeks old, the means of transport was boda-boda, which is risky for a child of that age”		

**Table 6:** Difficulties encountered by parents concerning treatment phase.

Phase	Frequency	Sample Comments
Casting	84	“The plaster was so heavy to carry the child and transport was difficult.” “Because I have to be careful that the urine can’t enter in the cast, so I have to buy pamper every time”
Tenotomy	28	“Because the child had so much pain and he cried for almost a week and I had sleepless nights, yet they were twins and I had to take care of the other one, yet also this one needed special attention”
Bracing	35	“Bracing was challenging because my son used to cry a lot and fight the time of bracing. Sometimes he would remove them. Also the 3 months of wearing them 24 h for 3 months, it made the right leg to be in a position that was abnormal. The braces made it abnormal.”
No challenging phases	11	
	Number of respondents mentioning at least one challenge: 152 Mean number of challenges mentioned: 1.1 (max = 3)	
“Casting: I couldn’t put the child down, I feared could spoil it. Tenotomy: child became very sick, cried a lot. I even had to call the facility but did not have money for transport. Bracing: I could not put the child on the back”		

## Data Availability

Data availability is restricted according to parameters of the participants’ informed consent. Data requests for research purposes can be made to the funding organization, MiracleFeet: https://www.miraclefeet.org/ and/or submitted to MEAL@MiracleFeet.org.

## Supplementary Materials

The following supporting information can be downloaded at: https://www.mdpi.com/article/10.3390/ijerph20146396/s1, Table S1: Brace dropout: possible mitigation strategies.
