# Supplementary material for: Exploring Bracing Adherence in Ponseti Treatment of Clubfoot: A Comparative Study of Factors and Outcomes in Uganda
Source: Int J Environ Res Public Health. 2023 Jul 19;20(14):6396. doi: 10.3390/ijerph20146396 (PMC10379221; doi:10.3390/ijerph20146396)
Supplement: Supplementary file 1 [file ijerph-20-06396-s001.zip › ijerph-2462410-supplementary.pdf]

Table S1. Brace Dropout: Possible Mitigation Strategies:

Problem	Potential strategy/action to mitigate
Long travel times to clinic/poor transportation options	• Prioritize families who have travelled very long distances to the clinic to give them enough time to return home. (Explain to other parents, to avoid tension in the waiting room)<br>• Provide hostel facilities for families to stay overnight.<br>• Establish more clubfoot clinics closer to patients.<br>• If parents are experienced and responsible with the brace, consider sending them home with an extra brace and instructions when to change to the bigger size.<br>• Mobile clinics - clinical outreaches to review brace patients closer to home.<br>• Remote brace reviews and encouragement by telephone (WhatsApp video)<br>• Train health centre staff closer to patients in brace checks only, under remote and in-person supervision visits of closest clubfoot clinic (Somalia model).<br>• Send new brace to family with local transport (bus, motor taxi etc.)
Long waiting times at clinic	• Ensure a well-organized clubfoot clinic with sufficient clinic supplies, ready for use.<br>• Ensure that clinic staff arrive in time to avoid parents having to wait longer than necessary.<br>• Ensure that parents can wait comfortably, with enough seating, organize activities/discussions/distractions in the waiting room.<br>• Organize a second clinic day, or a special brace checkup day.<br>• Lobby for more (trained) staff.
Competing financial priorities for family. High travel costs	• Transport subsidies—have clear guidelines, especially for parents covering long distances, very poor and at risk for dropout.<br>• Ensure good accountability for the transport subsidy system
Parents are not contactable/family moves	• For clinics who use CAST, have list of who has an appointment for that clinic day.<br>• Input as many phone numbers as possible in CAST/ your patient record system (parents/guardians, relatives and e.g., neighbour, local leader, Village Health Worker).<br>• Check phone numbers every visit.<br>• Enter physical address in CAST/patient record system, with landmarks how to get there.<br>• Call parents preferably immediately after a missed appointment and motivate them to come back/reschedule. It will help if the caller is someone the parents know/trust and does not blame them.<br>• Give the parents contact number(s) of the clinic, so that they can call you!<br>• Ask families if they have any plans to move or travel when scheduling the next appointment.<br>• When a family moves to a different area, refer them to a clubfoot clinic close to the new address (in writing).<br>• Have a list/poster with all clubfoot clinics in the country so that parents can see where else clinics are.<br>• If family moves, send information to the new clinic.
Parents have difficulty understanding or following the brace protocol	**Remember: The parents are the experts on their children and their family. They are vital to treatment success and should be fully informed and encouraged: good outcomes are not possible without their support. Listen to them and respect them as important members of the treatment team.**<br>• Build up a good relationship and show empathy. Understand their challenges. Take enough time, it is a good investment and prevents dropouts!<br>• Recruit (volunteer) parent advisors for every clubfoot clinic day.

Table S1. Brace Dropout: Possible Mitigation Strategies:

	• Organize training of providers and parent advisors on parent education and interaction. There are different training options (the CEDA program through GCI, MiracleFeet parent education training)<br><br>**Research shows that the highest bracing dropout occurs immediately after the first brace fitting.**<br>*Educate families:*<br>• Explain the **whole** Ponseti treatment (casting *and* bracing) at the very first appointment and remind them that bracing is a critical part of treatment at every opportunity. Explain what a relapse is and how often it occurs. Explain it in a way they understand; Explain why the brace looks the way it is and why it does not work when the shoes are not connected by the bar.<br>• Put up **posters** in your clinic about bracing.<br>• Ensure that parent knows how to put on the brace by letting them show you before they go home.<br>• Provide parents with a **brochure** with all steps of the treatment, including the bracing. They can show it at home to their spouse and relatives, so that the entire family understands and supports the treatment and the bracing.<br>• Coach and counsel the parents on challenges to expect and how to mitigate -critically important that this is done at first bracing visit. Encourage parents (using parent advisors, videos, written materials) that as long as the brace is not causing blisters or blue toes, the child will get used to the brace if it stays on. Frequently removing it will teach the child to continue to cry in order to have it removed.<br>• Build formal parent education and health information into routine clinic days, where old and new parents can meet and interact.<br>• Research shows that **most people immediately forget about 80% of the medical information given to them.** And **half of the information** that people do remember **is not correct.** Use the Teach-Back method, to ensure that parents have understood what you told them. In Teach-Back you ask parents to explain in their own words what they need to know or do. See next page.<br>*Support families*<br>• Start a parent/buddy group, where parents can exchange ideas/tips how to deal with treatment/bracing issues. This can be done in person or online (e.g. through WhatsApp or Facebook groups). A volunteer parent who has gone through the experience of the treatment process may be of great help for other parents.<br>• Supportive SMS messages<br>• Parent support staff to phone families within their first week after receiving a brace to check on them and help troubleshoot.<br>*Motivate/praise families.*<br>• Reward system for completing certain phases of treatment (e.g. First brace visit, 12 months of bracing, 2 years of bracing etc.)- such as giving honor in the clubfoot clinic, giving a small gift, taking a photo to mark the occasion etc.
Lack of family support	• Have parent advisors phone family members/partners to help them understand the treatment process.<br>• Training, materials, and transportation support for staff/volunteers/village health workers to make home visits to explain importance of treatment.<br>• Acknowledge and address any role of religious leaders in dissuading families from seeking treatment and discuss with these leaders directly.<br>• Encourage in-person/online peer groups to share experiences and successful approaches other mothers or parents have used to gain family acceptance/support.

Table S1. Brace Dropout: Possible Mitigation Strategies:

	• Written material from the clinic to take home and show family (extra materials for extended family/neighbours/community)<br>• Inform parents about the rights of a child to access treatment.<br>• Use social media to build a community of parents who support and encourage one another.
Parent forgets appointment	• Have a specific person/person who knows the parent to follow up with parents as soon as they have missed an appointment to reschedule.<br>• Use CAST tools to track who should be in the clinic that day.<br>• Automated SMS appointment reminders shortly before the next appointment<br>• Parent should never be blamed for missing an appointment, for a relapse or any other issue. Supportive attitudes are critical to keep families in treatment
Clinic staff in need of support and mentorship	Clinic staff need to be visited regularly by well-trained supportive supervisors who offer a listening ear, mentor, and give advice on how to improve clinic organization and clinic flow. Supportive supervisors also report back to country coordinators, who can lobby if needed for increase of staff, transport subsidies etc.

How to Use the Teach Back Method

- Start with the most important message.
- Limit to 2 – 4 key points
- Use plain simple language.
- Let the parent Teach-Back
- Rephrase the message until the parent demonstrates clear understanding.
- Once you are satisfied that the parent has understood the information, you can give the next key points and ask for Teach-Back again.

Some examples of Teach Back questions are:
"I know we have just talked about a lot, and I want to make sure that I didn't forget anything. Can you tell me what you have heard in your own words?"

"Just to be safe that I explained things well: can you tell me in your own words for what possible problems with the brace you need to come back to the clinic?"

"When you are explaining to family members at home, what would you tell them about what to do if the baby is crying in the brace?"

Watch this video and the method will become clear: https://www.youtube.com/watch?v=cllXBnHBiD4.
